# Supplementary material for: Root and Leaf Anatomy, Ion Accumulation, and Transcriptome Pattern under Salt Stress Conditions in Contrasting Genotypes of Sorghum bicolor
Source: Plants (Basel). 2023 Jun 21;12(13):2400. doi: 10.3390/plants12132400 (PMC10346968; doi:10.3390/plants12132400)
Supplement: Supplementary file 1 [file plants-12-02400-s001.zip › Supplementary Table 1.docx]

**Supplementary Table 1.** Paired-end reads used for reference based read mapping.

| **Sample** | **No. of filtered reads** | **Total no. of bases** | **Data in giga bases** |
| --- | --- | --- | --- |
| SS (ICSR-56) | 3,93,37,992 | 5,93,40,97,631 | 5.9 |
| SSN | 4,69,01,412 | 7,07,53,85,212 | 7.1 |
| ST (CSV-15) | 4,07,63,606 | 6,14,90,44,580 | 6.1 |
| STN | 3,78,04,841 | 5,70,28,89,085 | 5.7 |
